# Irradiation of silver and agar/silver nanoparticles with argon, oxygen glow discharge plasma, and mercury lamp

**DOI:** 10.1186/2193-1801-3-443

**Published:** 2014-08-19

**Authors:** Mahmoud M Ahmad, Essam A Abdel-Wahab, A A El-Maaref, Mohammed Rawway, Essam R Shaaban

**Affiliations:** Physics Department, Faculty of science, Al-Azhar University-Assuit branch, Assuit, Egypt; Botany and Microbiology Department, Faculty of science, Al-Azhar University-Assuit branch, Assuit, Egypt

**Keywords:** Silver nanoparticles, Glow discharge plasma, UV light irradiation, Antibacterial activity

## Abstract

The irradiation effect of argon, oxygen glow discharge plasma, and mercury lamp on silver and agar/silver nanoparticle samples is studied. The irradiation time dependence of the synthesized silver and agar/silver nanoparticle absorption spectra and their antibacterial effect are studied and compared. In the agar/silver nanoparticle sample, as the irradiation time of argon glow discharge plasma or mercury lamp increases, the peak intensity and the full width at half maximum, FWHM, of the surface plasmon resonance absorption band is increased, however a decrease of the peak intensity with oxygen glow plasma has been observed. In the silver nanoparticle sample, as the irradiation time of argon, oxygen glow discharge plasma or mercury lamp increases, the peak intensity of the surface plasmon resonance absorption band is increased, however, there is no significant change in the FWHM of the surface plasmon resonance absorption band. The SEM results for both samples showed nanoparticle formation with mean size about 50 nm and 40 nm respectively. Throughout the irradiation time with the argon, oxygen glow discharge plasma or mercury lamp, the antibacterial activity of several kinds of Gram-positive and Gram-negative bacteria has been examined.

## Introduction

Due to their unique properties, metal nanoparticles are attracted a great interest of experimental and theoretical investigations (Amoruso et al. 2005; Link and El-Sayed 2000; Richardson et al. 2009; Shalaev 2002). Controlling of metalic nanoparticles geometry has found special interset since this allows tuning optical properties that are not present in bulk materials (Ahmadi et al. 1996). The tunability of the plasmon position and its charachterstics such as Full width at half maximum (FWHM), and peak intensity makes the nanoparticles attractive for several applications (Cobley et al. 2009; McFarland and Van Duyne 2003). The mainpulation of nanoparticles can be achieved with laser, UV-light as well as with plasma sources (Hou et al. 2013; Mafune et al. 2000; Zhen et al. 2013). In principle, irradiation with a given light source excites and heats nanoparticles of certain sizes or/and shapes and leads to diffusion and evaporation of surface atoms. Thus, tuning the plasmon position and its charachterstics of the nanoparticles can be accomplished. In the present experiment, silver and agar/silver nanoparticles were prepared using chemical reduction method (Pillai and Kamat 2004). The prepared samples have been irradiated with argon, oxygen glow discharge plasma sources, and mercury lamp at different time periods. Spectrophotometric measurements were carried out to follow the irradiation process and to characterize the optical properties of the resultant silver and agar/silver nanoparticles. Finally, the resultant nanoparticles samples have been examined for antibacterial activity against various types of Gram-positive and Gram-negative bacteria, which are necessary in order to fully evaluate its possible use as a new bactericidal material.

## Methods

### Synthesis

The silver nanoparticle samples have been prepared by using chemical reduction method. All solutions of reacting materials have been prepared in distilled water. Silver nitrate AgNO_3_ and trisodium citrate C_2_ H_5_O_7_Na_3_ of analytical grade purity were used as starting materials without further purification. In the present procedure 150 mL of 1 mM AgNO_3_ was heated to boiling and 15 mL of 1% trisodium citrate was added drop by drop to the solution until its color change to pale yellow. Then it was removed from the heating plate and stirred until cooled to room temperature and it kept in dark place. The agar/Silver nanoparticles sample has been prepared by adding 0.1 gm of agar powder to 10 mL of silver nanoparticles solution and stirred for two minutes at room temperature.

For SEM analysis, samples are prepared by depositing a drop of colloidal solution on a carbon coated copper SEM holder and drying at room temperature. Absorption spectra were recorded at room temperature using Jasco-670 double beam spectrometer.

### Discharge plasma setup and Irradiation procedures

**Figure 1:**
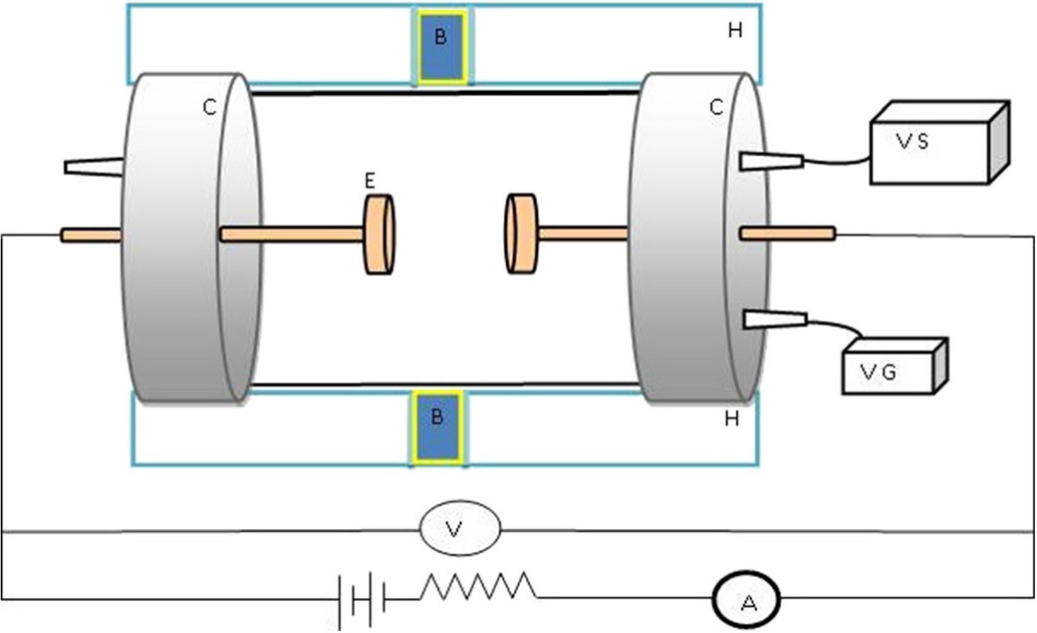
**Schematic diagram of an electric discharge cell.** Where E is two copper circular plane electrodes (0.2 cm thickness and 5 cm diameter), C is the reaction chamber (cylindrical Pyrex glass of 7 cm diameter and 15 cm length with 2 cm gap spacing, VS is the vacuum system, VG is the vacuum gauge, H is the sample holder, and B is cuvette place holder.

In order to setup argon, oxygen glow discharge plasma sources, two copper circular plane electrodes are used. The two electrodes are centered in the reaction chamber axes. The gas has injected into the reaction chamber through the side flange. The reaction chamber was evacuated up to 10^−3^ mmHg before the gas inlet. The gas pressure has controlled using vacuum system and gauges to 0.11 mmHg and kept constant during the measurement procedure. The discharge voltages of the argon and oxygen plasma were 248 and 358 volt respectively. More details of the plasma source setup can be found in (Shaaban et al. 2013). Figure 1 shows a schematic diagram of the discharge plasma setup. The strong emission spectral lines of argon, oxygen glow discharge plasma sources and mercury light source are listed in Table 1 (Reader et al. 1996; Bacławski and Musielok 2008).

**Table 1:** **The strongest emission lines in nm of argon, oxygen glow discharge plasma sources and mercury light source (Bacławski and Musielok** 2008 **; Reader et al.** 1996 **)**

Oxygen lines (nm) O I	Mercury lines (nm) Hg I	Argon lines (nm) Ar I
777.337	253.652	696.543
645.499	296.728	706.722
725.436	302.150	714.704
615.727	313.155	727.294
715.670	334.148	738.398
926.387	365.015	750.387
948.289	404.656	763.511
949.794	407.783	772.376
950.560	435.833	794.818
949.271	546.074	826.452
948.743	576.960	842.465
	579.066	852.144
		866.794
		912.297
		922.450

Six samples of the same volume 3 mL of silver and agar/silver nanoparticles have been irradiated with argon, oxygen glow discharge plasma sources, and mercury lamp at different time periods. Spectrophotometric measurements were carried out to follow the irradiation process and to characterize the optical properties of the resultant silver and agar/silver nanoparticles.

### Antibacterial procedures

The disc diffusion assay method was used to study the antibacterial activity of the synthesized nanoparticle samples (NCCLS 1993). All the glassware, media and reagents used were sterilized in an autoclave at 121°C for 20 min.

The antibacterial activity of nanoparticle samples was evaluated against some of Gram positive (*Bacillus cereus, Bacillus subtilis, Micrococcus roseus, Staphylococcus aureus and Streptococcus sp.*) and Gram negative bacteria (*E.coli, Klebsiella pneumoniae, Proteus vulgaris, Pseudomonas aeruginosa and Serratia marcescens*). Bacterial suspension was prepared by growing a single colony overnight in nutrient broth and by adjusting the turbidity to 0.5 McFarland standards (Kora et al. 2009).

Media plates were inoculated with this bacterial suspension. The sterile filter-paper disks (Whatman filter paper no.1) of 6 mm were impregnated with nanoparticle samples solution and placed on the surface of the media inoculated with bacterial species. These plates were incubated at 37°C for 24 h and the zone of inhibition (ZOI) was measured.

## Results and discussions

**Figure 2:**
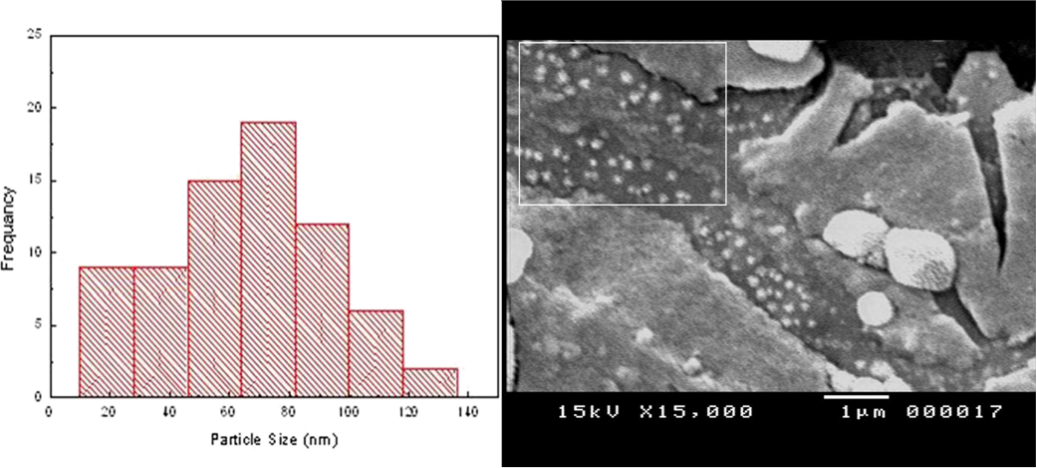
**SEM image and particle size histogram of silver nanoparticles.**

**Figure 3:**
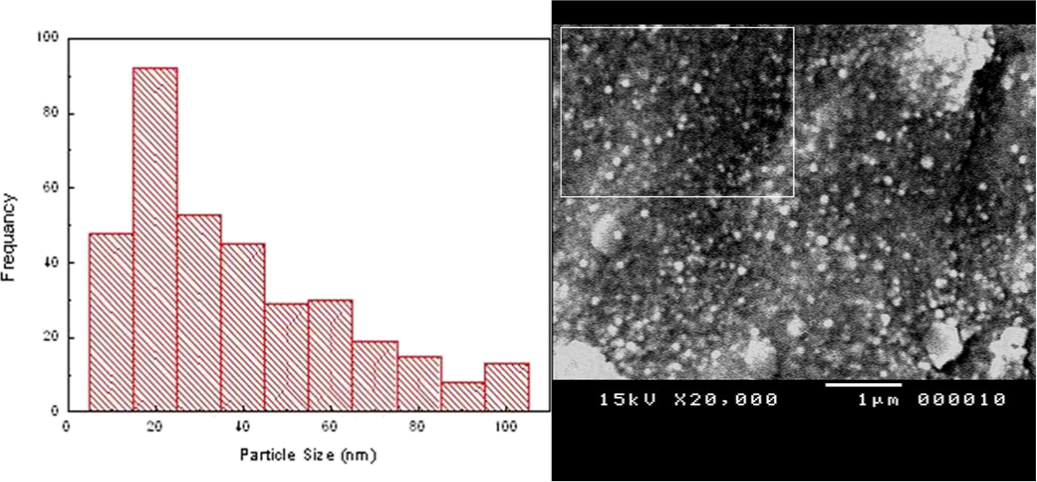
**SEM image and particle size histogram of agar/silver nanoparticles.**

SEM analysis has been performed in order to observe morphology of the synthesized samples. Figures 2 and 3 show SEM images and the corresponding size distributions of silver and agar/silver nanoparticle samples respectively. From SEM images the silver and agar/silver nanoparticles are spherically shaped with mean size about 50 nm and 40 nm respectively. The effect of agar powder on the synthesized silver nanoparticles states as controller of nucleation as well as stabilizer.

Measurements of UV–vis spectrophotometer show the expected surface plasmon resonance SPR peak of silver nanoparticles. In the UV–vis spectra a single strong peak with a maximum around 424 nm is observed of silver nanoparticle samples, which corresponds to the typical SPR of conducting electrons of the surface of silver nanoparticles. In agar/silver nanoparticle samples a single strong peak with a maximum around 428 nm has been detected. This shift of peak position of the SPR band between the two samples is due to the sensitivity of SPR to the shape, size, and interaction of the particle with the medium and local refractive index.

It is observed that, there are no peaks located around 335 and 560 nm, which indicate to the complete absence of nanoparticle aggregation (Mohan et al. 2007; Kora et al. 2009).

**Figure 4:**
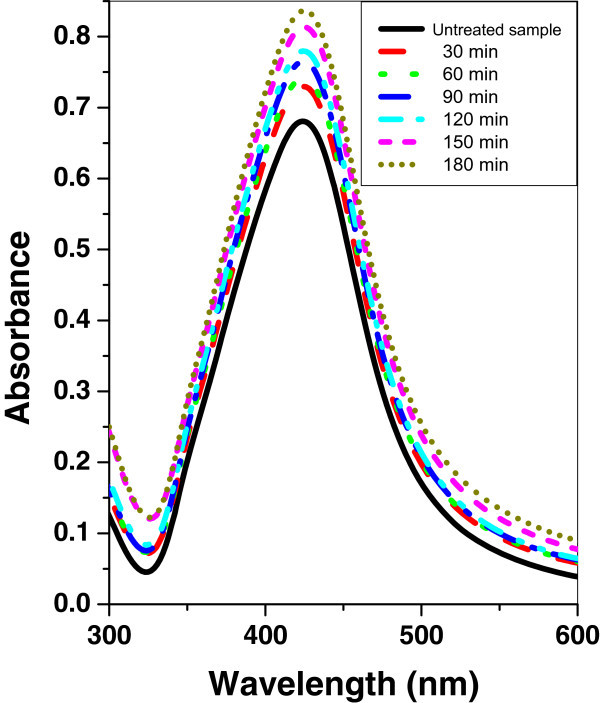
**UV–vis absorbance spectra of silver nanoparticle sample before and after irradiation with argon plasma at different time periods.**

**Figure 5:**
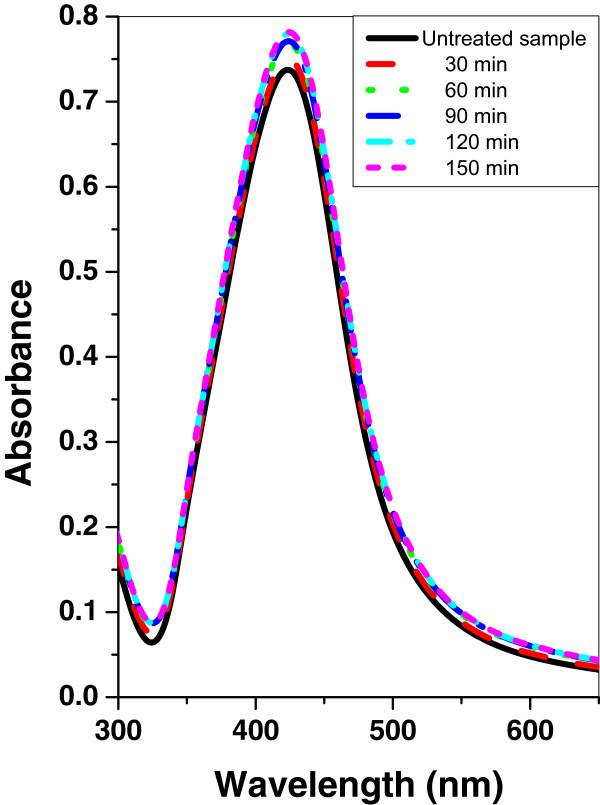
**UV–vis absorbance spectra of silver nanoparticle sample before and after irradiation with oxygen plasma at different time periods.**

**Figure 6:**
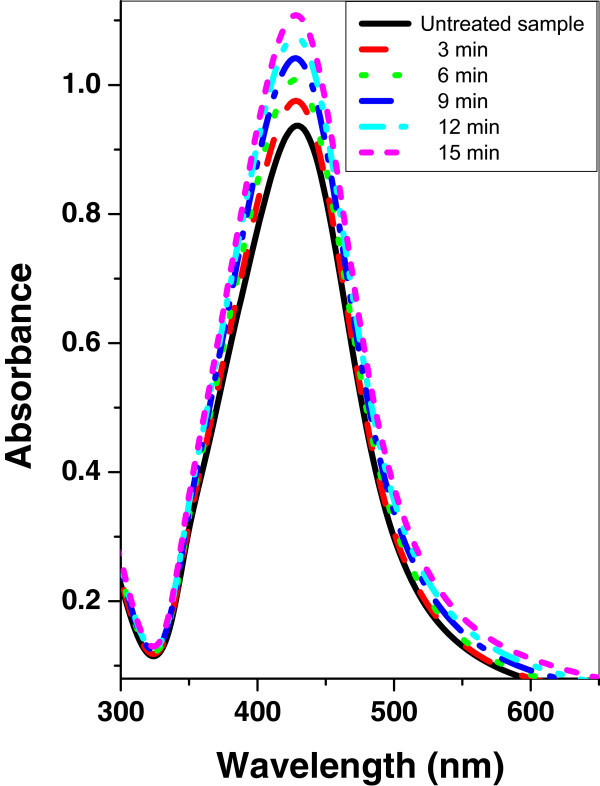
**UV–vis absorbance spectra of silver nanoparticle sample before and after irradiation with mercury lamp at different time periods.**

Figures 4, 5, and 6 show the absorption spectrum of silver nanoparticle samples irradiated with argon, oxygen, and mercury lamp at different time intervals respectively. It is shown that, in the case of silver nanoparticle sample as the irradiation time of argon, oxygen glow discharge plasma or mercury lamp increases, the peak intensity of the SPR absorption band is increased. However, there is no significant change of the FWHM at the SPR absorption band.

**Figure 7:**
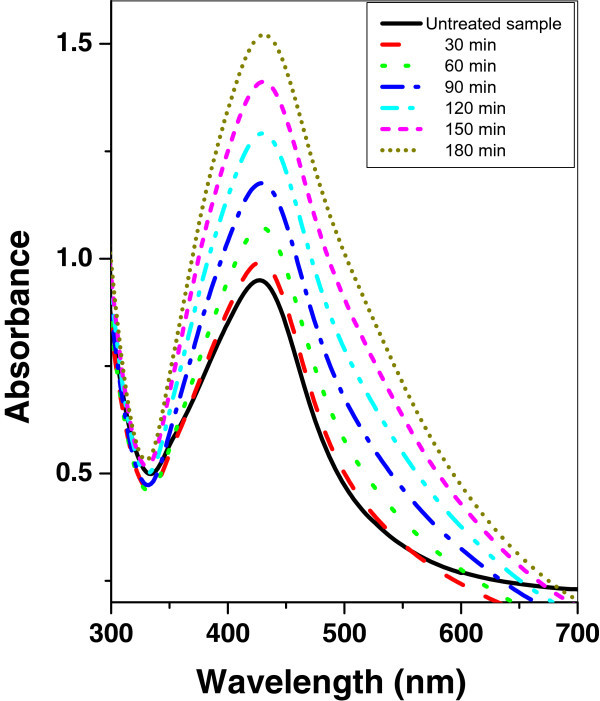
**UV–vis absorbance spectra of agar/silver sample before and after irradiation with argon plasma at different time periods.**

**Figure 8:**
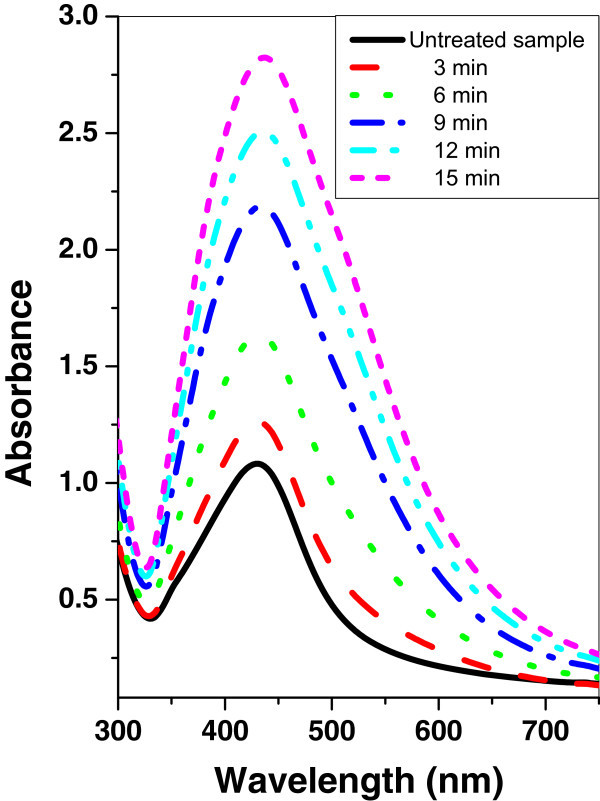
**UV–vis absorbance spectra of agar/silver nanoparticle sample before and after irradiation with mercury lamp at different time periods.**

**Figure 9:**
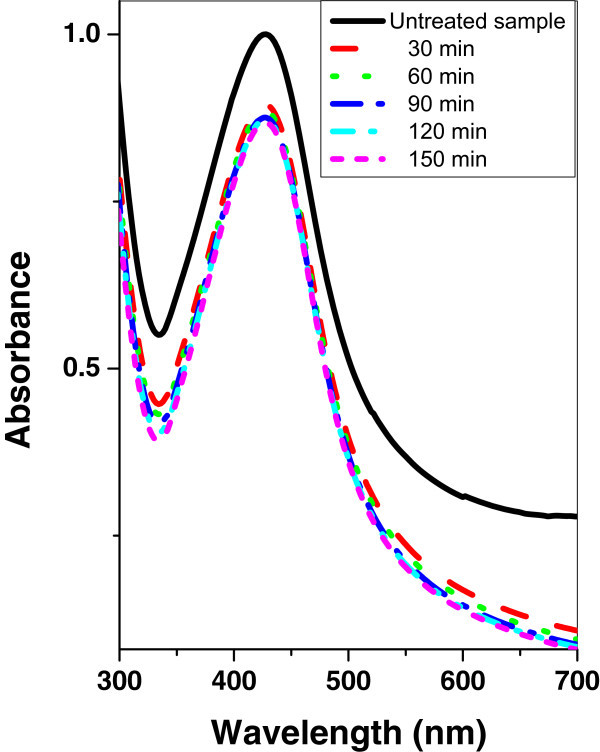
**UV–vis absorbance spectra of agar/silver nanoparticle sample before and after irradiation with oxygen plasma at different time periods.**

Figures 7, 8, and 9 show the absorption spectrum of agar/silver nanoparticle samples irradiated with argon, oxygen, and mercury lamp at different time intervals respectively. It is shown that for agar/silver nanoparticle sample as the irradiation time of argon glow discharge plasma or mercury lamp increases, the peak intensity and the FWHM of the SPR absorption band are increased. However a decrease of the peak intensity with oxygen glow discharge plasma is observed and the FWHM remains constant.

**Figure 10:**
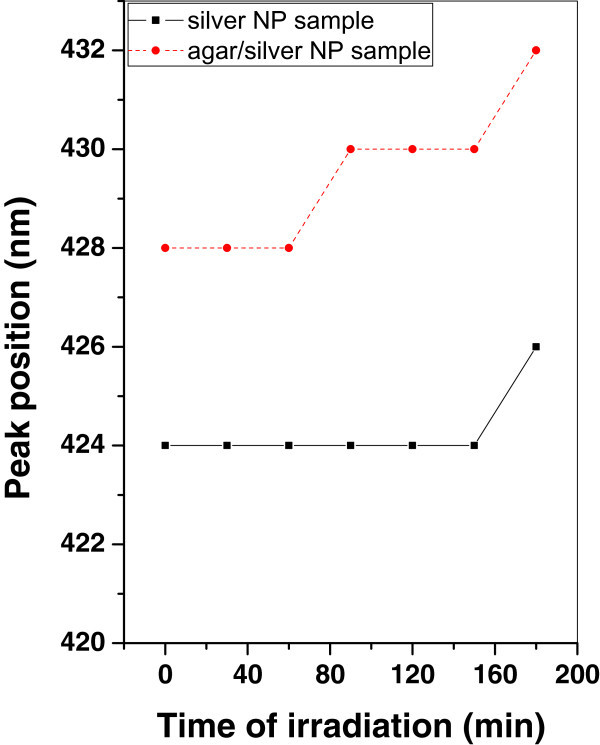
**Variation of SPR peak position of silver and agar/silver samples after irradiation with argon plasma.**

**Figure 11:**
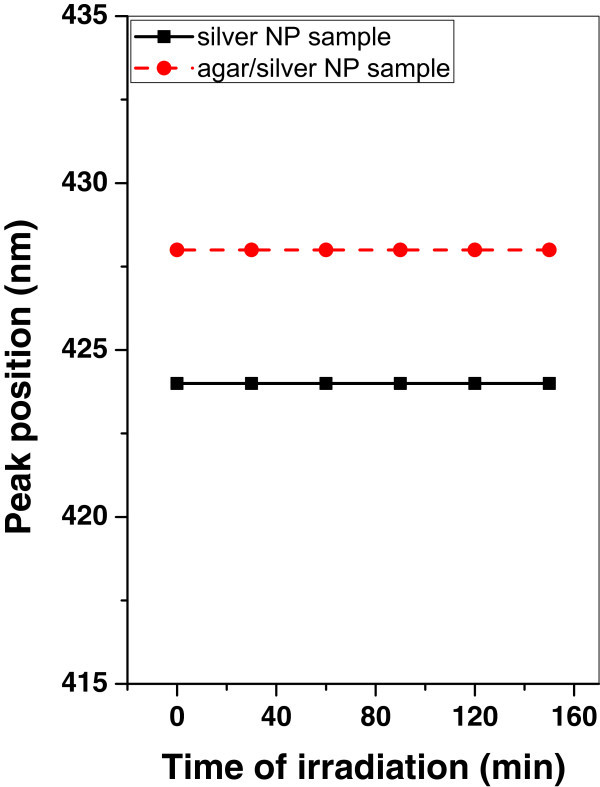
**Variation of SPR peak position of silver and agar/silver samples after irradiation with oxygen plasma.**

**Figure 12:**
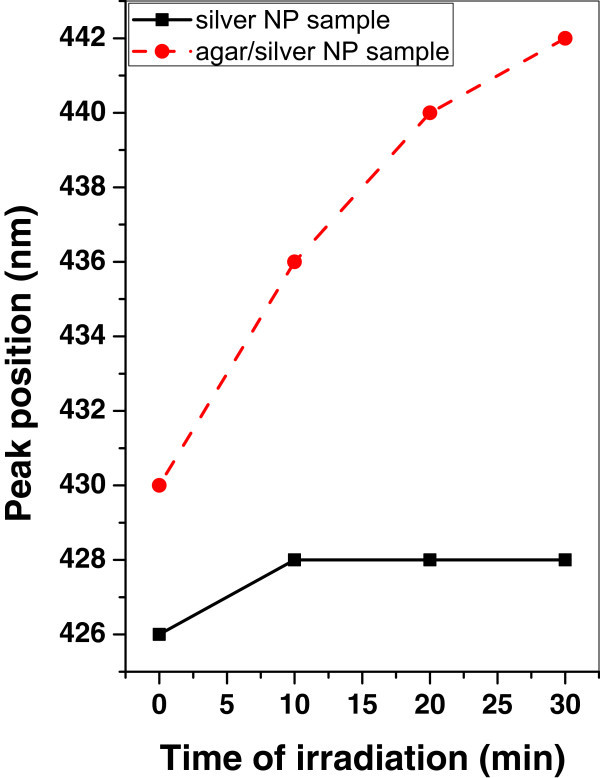
**Variation of SPR peak position of silver and agar/silver samples after irradiation with mercury lamp.**

Variation of SPR peak position of silver and agar/silver nanoparticle samples after irradiation with argon, oxygen plasma and mercury lamp have been plotted in Figures 10, 11, and 12 respectively. It is shown that, the SPR peak position remains constant in the case of silver nanoparticle sample irradiated with argon plasma, however a stepwise increment is observed in the case of agar/silver nanoparticle sample.

When both samples are irradiated with oxygen plasma, the SPR peak position remains constant.

It is observed that, the SPR peak position is increased for agar/silver nanoparticle sample after irradiation with mercury lamp. However there is no significant change of SPR peak position of silver nanoparticle sample.

**Figure 13:**
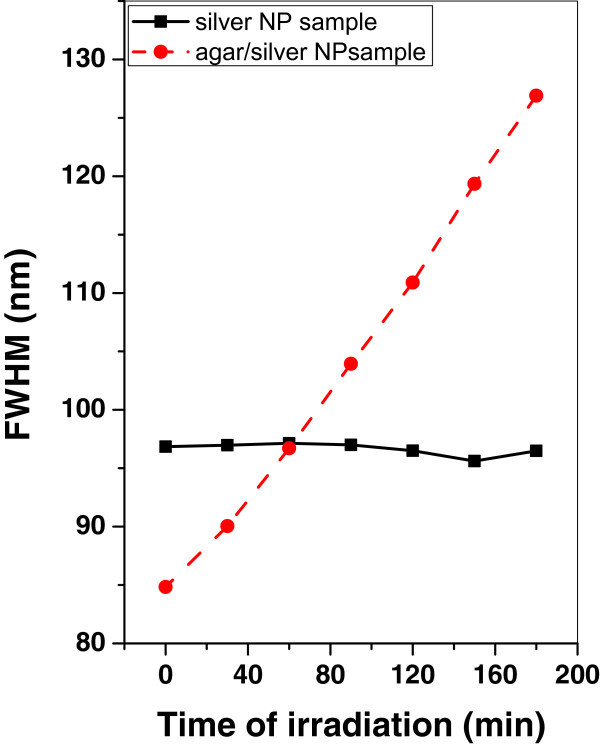
**Variation of FWHM of silver and agar/silver samples after irradiation with argon plasma.**

**Figure 14:**
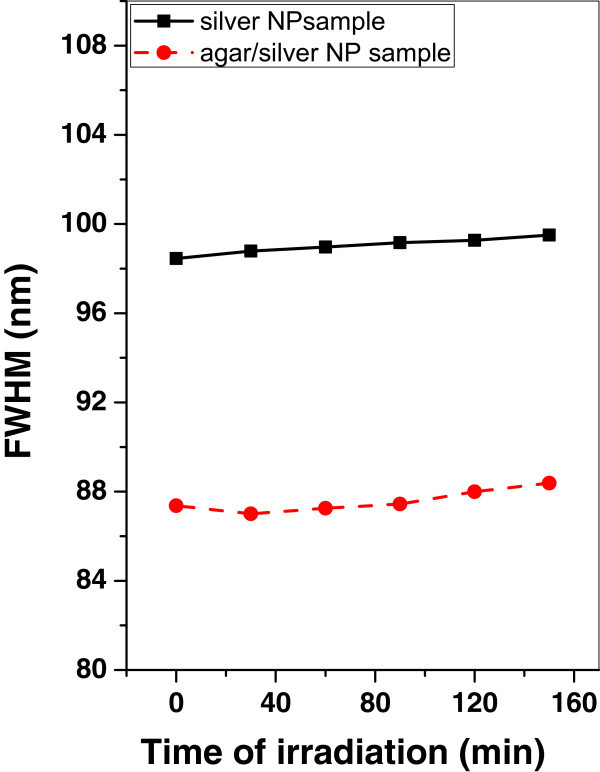
**Variation of FWHM of silver and agar/silver samples after irradiation with oxygen plasma.**

**Figure 15:**
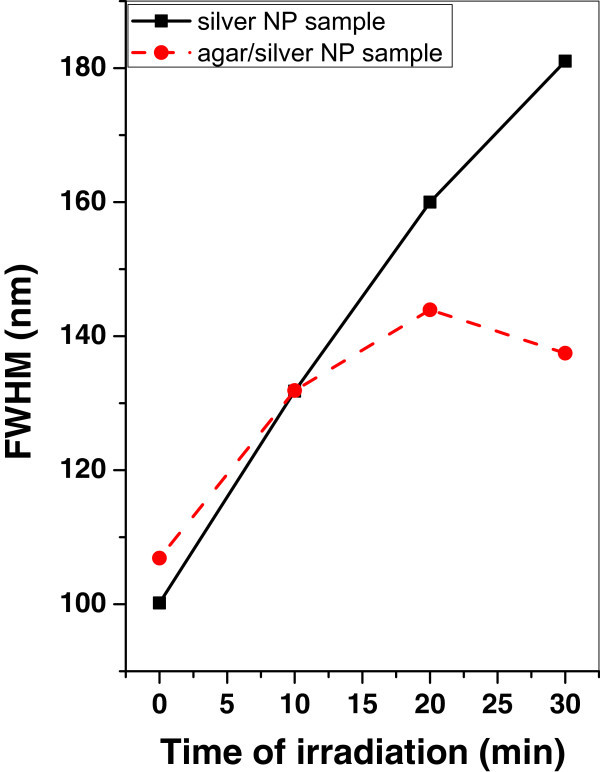
**Variation of FWHM of silver and agar/silver samples after irradiation with mercury lamp.**

Figures 13, 14, and 15 show the variation of the FWHM of silver and agar/silver nanoparticle samples after irradiation with argon, oxygen plasma and mercury lamp respectively. It is observed that, there is no change of FWHM for silver nanoparticle sample irradiated with argon plasma however the FWHM is increased rapidly for agar/silver nanoparticle sample. In the case of oxygen plasma irradiation the FWHM remains constant for both samples.

After irradiation with mercury lamp the FWHM increases rapidly for silver nanoparticle sample; however there is a hysterics behavior of agar/silver nanoparticle sample.

The above results are discussed in terms of a mechanism in which, agar consists of a mixture of agarose and agaropectin. It is composed of alternating 1,3-linked d-galactose and 1,4-linked 3,6 anhydro-l galactose units (Labropoulos et al. 2002). It has the ability to form reversible gels simply by cooling a hot aqueous solution.

It is well known that, polysaccharide contains of hydroxyl, acetyl, carbonyl and carboxylic functional groups. This disaccharide can be substituted by sulfate esters and methoxyl, and may also carry pyruvic acid residues (Duckworth and Yaphe 1971). The type, amount, and location of these substitutes strongly affect the physical properties of the gel and therefore, its functionality (Freile-Pelegrin and Murano 2005).

Based on these facts, it can be inferred that both hydroxyl and carbonyl groups of agar are involved in the synthesis of agar/silver nanoparticle sample and effectively help in capping the surface of nanoparticles. The variations in the shape and peak position of the hydroxyl and carboxylate groups using FTIR have been reported (Guerrero et al. 2014). Also silver nanoparticles can synthesized using another polysaccharide i.e. gum Acacia (Mohan et al. 2007), gum kondagogu (Kora et al. 2010) and gum Arabia (Gils et al. 2010).

Throughout the irradiation time with the argon, oxygen glow discharge plasma or mercury lamp, the antibacterial activity of several kinds of bacteria has been examined. Table 2 shows diameter of bacterial inhibition (clear zone) in mm. Bacterial strains number 1, 2, 5, 9 and 10 are Gram-positive bacteria while bacterial strains number 3, 4, 6, 7 and 8 are Gram-negative bacteria.

**Table 2:** **Diameter of bacterial inhibition zone (clear zone) in mm**

Sample name		Untreated silver NP (A)	Untreated agar/silver NP (B)	Treated (A) with argon plasma for 180 min	Treated (B) with argon plasma for 180 min	Treated (A) with oxygen plasma for 150 min	Treated (B) with oxygen plasma for 150 min	Treated (B) with mercury lamp at for 40 min	Treated (A) with mercury lamp for 15 min	Treated (B) with mercury lamp for 15 min
No.	***Bacteria name***									
*1*	*Bacillus cereus*	8	8	10	9	8	8	8	8	8
*2*	*Bacillus subtilis*	9	10	9	9	8	8	8	8	8
*3*	*E. coli*	11	14	12	13	11	11	10	11	11
*4*	*Klebsiella pneumoniae*	15	10	10	10	10	10	10	12	10
*5*	*Micrococcus roseus*	16	11	10	10	11	10	10	11	10
*6*	*Proteus vulgaris*	11	8	9	15	9	9	8	8	8
*7*	*Pseudomonas aeruginosa*	12	11	14	12	16	14	12	14	12
*8*	*Serratia marcescens*	9	10	9	9	9	9	9	10	9
*9*	*Staphylococcus aureus*	17	15	12	11	12	12	11	12	10
*10*	*Streptococcus sp.*	15	18	13	13	17	14	13	14	12

## Conclusions

In summary, the irradiation effects of argon, oxygen glow discharge plasma, and mercury lamp on silver and silver/agar nanoparticle samples are studied and compared. The tunability of the SPR position and its characteristics such as FWHM and peak intensity has been investigated. Therefore, the choice of suitable light source leads to controlling the SPR characteristics.

In the present process, glow discharge plasma and mercury lamp irradiation could have high potentials to enhance photochemical reduction method. The irradiation procedure is simple and reproducible and it can be operated at different glow discharge plasma conditions.

The virgin and treated nanoparticles samples exhibited strong antibacterial activity against both the Gram-positive and Gram-negative bacteria. Therefore, the resulting silver and agar/silver nanoparticles samples with antibacterial activity could have high potentials for many applications such as an antibacterial food packaging and a biomedical application such as wound dressings. However, actual applications of antibacterial nanoparticles require further studies focused on the potential health-hazard of such nanoparticles included products.
